# The Treatment of Placenta Prævia

**Published:** 1901-04-13

**Authors:** 


### THE TREATMENT OF PLACENTA PRÆVIA.

Dr. Ranken Lyle,[1] obstetric physician to the Newcastle Lying-in Hospital, says that for purposes of treatment most cases of placenta prævia may be divided into three classes. First, cases of incomplete placenta plævia in which the first stage of labour is fairly well advanced; rupture of the membranes and the application of a tight abdominal binder will here be usually found a sufficient treatment, but in a rare case this may not check the hæmorrhage, when version (if necessary) should be performed, and a foot brought down, leaving the subsequent delivery to nature, with the exception of course of slight traction on the foot should hæmorrhage continue. Secondly, cases of complete or incomplete placenta prævia in which the os uteri is sufficiently dilated to admit two fingers. It is in the treatment of these cases that so much difference of opinion exists, some recommending the rapid evacuation of the uterus, irrespective of the injury which may be done to the mother's soft parts, and indeed of the risk to the patient's life; while others recommend the separation of the placenta from the uterine wall with the finger, a proceeding which many consider not only fatal to the child, but exceedingly dangerous to the mother on account of the risk of septic infection. The treatment which has been adopted at the Rotunda Hospital for many years with marked success is as follows: In cases of central or complete placenta prævia, the placenta is perforated with the fingers, version (if necessary) is performed, and a foot brought down and the subsequent delivery is left to nature, unless, of course, the continuance of hæmorrhage should necessitate slight traction on the foot. In cases of incomplete placenta prævia the treatment is the same with the exception of rupture of the membranes being done instead of perforation of the placenta. Thirdly, cases of placenta prævia in which the os uteri is not sufficiently dilated to admit two fingers. These cases are extremely rare and should be converted into cases of the first or second class by plugging the vagina tightly with boiled cotton wool and the application of a tight abdominal binder until labour has advanced sufficiently to dilate the cervix, and then treat accordingly. During the ten years 1889-99 seventy-four cases of placenta prævia were treated in the Rotunda Hospital. There were four maternal deaths.

[1] Brit. Med. Journ., April 6.

